# Synovial expression of IL-15 in rheumatoid arthritis is not influenced by blockade of tumour necrosis factor

**DOI:** 10.1186/ar1871

**Published:** 2005-12-28

**Authors:** Sofia Ernestam, Erik af Klint, Anca Irinel Catrina, Erik Sundberg, Marianne Engström, Lars Klareskog, Ann-Kristin Ulfgren

**Affiliations:** 1 Department of Rheumatology, Karolinska University Hospital, S-141 86 Stockholm, Sweden; 2 Department of Medicine, Rheumatology Unit, Karolinska Institutet/Karolinska University Hospital, Stockholm, Sweden; 3 Department of Women and Child Health, Pediatric Rheumatology Research Unit, Karolinska Institutet/Karolinska University Hospital, Stockholm, Sweden

## Abstract

Blockade of tumour necrosis factor (TNF) is an effective treatment in rheumatoid arthritis (RA), but both non-responders and partial responders are quite frequent. This suggests that other pro-inflammatory cytokines may be of importance in the pathogenesis of RA and as possible targets for therapy. In this study we investigated the effect of TNF blockade (infliximab) on the synovial expression of IL-15 in RA in relation to different cell types and expression of other cytokines, to elucidate whether or not IL-15 is a possible target for therapy, independently of TNF blockade. Two arthroscopies with multiple biopsies were performed on nine patients with RA and knee-joint synovitis before and after three infusions of infliximab (3 mg/kg). Synovial biopsies were analysed with immunohistochemistry for expression of IL-15, TNF, IL-1α, IL-1ß and IFN-γ, and for the cell surface markers CD3, CD68 and CD163. Stained synovial biopsy sections were evaluated by computerized image analysis. IL-15 expression was detected in all synovial biopsies taken at baseline. After infliximab therapy, the expression of IL-15 was increased in four patients and reduced in five. Synovial expression of IL-15 was not correlated with any CD marker or with the presence of any other cytokine. Synovial cellularity was decreased after 8 to 10 weeks of treatment with a significant reduction of the CD68-positive synovial cells, whereas no significant change was seen in the number of CD3-positive T cells and CD163-expressing macrophages. The number of TNF-producing cells in the synovial tissue at baseline was correlated with a good response to therapy. Thus, in this study the synovial expression of IL-15 in RA was not consistently influenced by TNF blockade, being apparently independent of TNF expression in the synovium. Consequently, we propose that IL-15 should remain as a therapeutic target in RA, regardless of the response to TNF blockade.

## Introduction

Blockade of tumour necrosis factor (TNF) in active rheumatoid arthritis (RA) is effective in reducing disease activity [1] and in stopping joint destruction [2]. However, both non-responders and partial responders to TNF blockade are frequent [1], indicating a considerable influence of other pro-inflammatory cytokines beside TNF in perpetuating inflammation in RA.

IL-15 has been identified as a pro-inflammatory cytokine of potential importance in the pathogenesis of RA [3].

In the synovial membrane of patients with RA, there is a substantial expression of IL-15, predominantly expressed in macrophages but also in fibroblast-like synoviocytes and endothelial cells [3-5]. IL-15 has been described as inducing chemotaxis and proliferation of T cells and acting through a cell-contact-dependent mechanism between memory T cells and macrophages [6]. IL-15 may also contribute to an increased production of the pro-inflammatory cytokines TNF, IFN-γ and IL-17 in T cells [7]. Taken together, these findings suggest that an IL-15-dependent pro-inflammatory feedback loop may be created in the inflamed synovium, in which IL-15 stimulates the production of TNF, IFN-γ and IL-17, which in turn stimulate the further production of IL-15, IL-8 and IL-6 in fibroblast-like synoviocytes [7,8].

An additional pivotal role of IL-15 as a link between the innate and adaptive immune system was recently suggested from the observation that activated T cells express Toll-like receptor 2, a co-stimulatory receptor that acts together with IL-15 in maintaining T cell activation [9].

Because IL-15 might have a central role in sustaining inflammation in RA, this cytokine has been considered as a potential therapeutic target in RA. Both a soluble fragment of the IL-15-receptor α-chain and an antagonistic IL-15 mutant/Fcγ2a fusion protein have been reported to suppress the development of collagen-induced arthritis in murine models [10,11]. These beneficial effects were accompanied by both a reduced production of TNF, IL-1ß, IL-6 and IL-17 in the inflamed joints of the treated animals and a decreased frequency of T cells reactive to anti-collagen II [11].

Finally, and most importantly, a phase I/II trial with an antibody blocking IL-15 given to RA patients was recently reported with promising results [12].

An important question in relation to the development of future therapies targeting IL-15 is how the expression of this cytokine will be affected by existing therapies, including TNF blockade. In this study we therefore investigated the effect of infliximab, a TNF-blocking antibody, on the synovial expression of IL-15 in relation to different cell types and the expression of other cytokines.

## Materials and methods

### Patients, treatment and clinical assessments

Nine patients (seven females, two males) with RA (seven positive for rheumatoid factor, and two negative) according to the American College of Rheumatology (ACR) criteria, and arthritis of the knee joint, were recruited for this study. All patients were assessed for disease activity at baseline and at three months, with the Disease Activity Score counted on 28 joints (DAS28). ACR response criteria were also used to record the result of the therapy. Functional capacity was recorded with the health assessment questionnaire. The median DAS28 at inclusion was 5.95 (range 4.83 to 7.91), despite treatment with methotrexate (7.5 to 20 mg per week). The dose of methotrexate was stable during the study and at least one month before the first arthroscopy. Four patients received prednisolone in an equally stable dose of not more than 10 mg per day. The median age was 57 years (range 25 to 69) and the median disease duration was 6 years (range 0.5 to 18). The median duration of the current episode of arthritis in the knee was 17.5 days (range 3 to 365, lacking data for one patient).

Three intravenous infusions of infliximab (3 mg/kg; Centocor B.V, Leiden, The Netherlands) were given in accordance with the recommended standard treatment protocol, with the first infusion given 1 to 21 days after the first arthroscopy and the subsequent infusions given after 2 and 6 weeks.

Informed consent was obtained from all patients, and the study was approved by the local ethics committee at the Karolinska University Hospital.

### Synovial biopsies, immunohistochemistry and computer-assisted image analysis

An arthroscopy with multiple biopsies of the knee joint was performed in all patients 1 to 21 days before the first infusion. A second arthroscopy was performed at 8 to 10 weeks (median 9 weeks) after the first infusion. Total knee joint replacement surgery was considered as a substitute for the second arthroscopy in patient no. 7. The same physician (EaK) performed all arthroscopies. At the first arthroscopy, biopsies were predominantly taken from areas of the synovial tissue with signs of maximal macroscopic inflammation, from the cartilage-pannus junction and from synovial villi. The biopsy site was documented photographically and at the second arthroscopy the biopsies were taken from the same area as the first biopsies. The biopsies were snap-frozen within 2 minutes in liquid isopentane and stored at -70°C until sectioned. Serial cryostat sections (7 µm) were fixed for 20 min with 2% (v/v) formaldehyde and stored at -70°C.

Several biopsies were taken to secure sufficient material. For each of the nine patients, the biopsy with the best morphology was selected for subsequent immunohistochemical staining. The staining was always performed in pairs before and after treatment infliximab, allowing a pairwise comparison.

Two anti-(human IL-15) mAbs were used, one neutralizing (B-E29) and one non-neutralizing (B-T15; both from Diaclone SAS, Besancon, France). The staining procedure has been described previously [13]. Biopsy specimens were also analysed for the presence of the cytokines IL-1α, IL-1ß, IL-2, IL-4, IFN-γ, TNF mAb1 and mAb11, and for the presence of the cell surface markers CD3, CD19, CD20, CD68 and CD163, as described previously [14]. In addition, for TNF a new neutralizing IgG1γ mAb, 2C8, was used (Biodesign, Maine, USA). Negative controls with isotype-matched IgG were included for each marker.

Two evaluators, blinded to the origin and order of the sections, performed a semi-quantitative analysis of the expression of cytokines and cell surface markers, considering the number of positive cells in the stained sections. TNF mAb1 and mAb11 and IFN-γ were scored with a semi-quantitative four-point scale as follows: 0 = no positive cells, 1 = 1 to 10 positive cells, 2 = 11 to 100 positive cells, and 3 = more than 100 positive cells [15]. Cell surface markers (CD3, CD68 and CD163) were scored with another semi-quantitative four-point scale: 0 = no infiltration, 1 = minimal infiltration, 2 = moderate infiltration, and 3 = marked infiltration [16]. In the blinded manner described, stained synovial biopsy sections were evaluated by computerized image analysis, in which the area of positive staining was expressed as a percentage of the total tissue area, for IL-15 neutralizing and non-neutralizing antibodies, IL-1α, IL-1ß and TNF mAb 2C8. Analysis of an entire tissue section typically involved 25 to 210 (median 70) microscope fields, corresponding to an area of 0.7 to 9.1 mm^2 ^(median 2.9 mm^2^) at a magnification of × 250.

To evaluate which cells were predominantly expressing IL-15, double staining was performed on samples from two of the patients (nos 3 and 5) for IL-15 neutralizing antibody and IL-15 non-neutralizing antibody, together with CD markers CD3, CD19/20, CD68 and CD163.

### Statistical analysis

A Mann-Whitney *U *test was used for the analysis of differences between groups. Wilcoxon's signed-rank test was used for the analysis of matched pairs. A Spearman rank correlation test was used for correlations between variables. *p *< 0.05 was considered statistically significant.

## Results

### Patients and response to treatment

The individual DAS28 values are presented in Table 1. Median DAS28 scores were reduced from 5.95 to 4.41 (*p *< 0.01), the median tender joint count from 10 to 3 (*p *< 0.05), the median swollen joint count from 14 to 2 (*p *< 0.01) and the median C-reactive protein from 34 mg/L to 19 mg/L (*p *= 0.08), and the median health assessment questionnaire improved from 1.63 to 1.38 (*p *< 0.05). Two patients fulfilled ACR 70%, one patient fulfilled ACR 50%, three patients fulfilled ACR 20% and three patients were non-responders according to the ACR criteria at clinical evaluation after treatment with three infusions of infliximab (Table 1).

### Immunohistochemical analysis

#### CD-markers

The results of the semi-quantitative analysis of the staining for CD markers are summarized in Table 2. A significant decrease in the CD68-positive synovial cells (*p *= 0.018) was observed in the second biopsies in comparison with the initial ones, whereas the numbers of CD3-positive T cells and CD163-positive activated macrophages were not significantly changed, although there was a trend towards a reduction.

#### Tissue distribution of staining for IL-15

**Figure 1:**
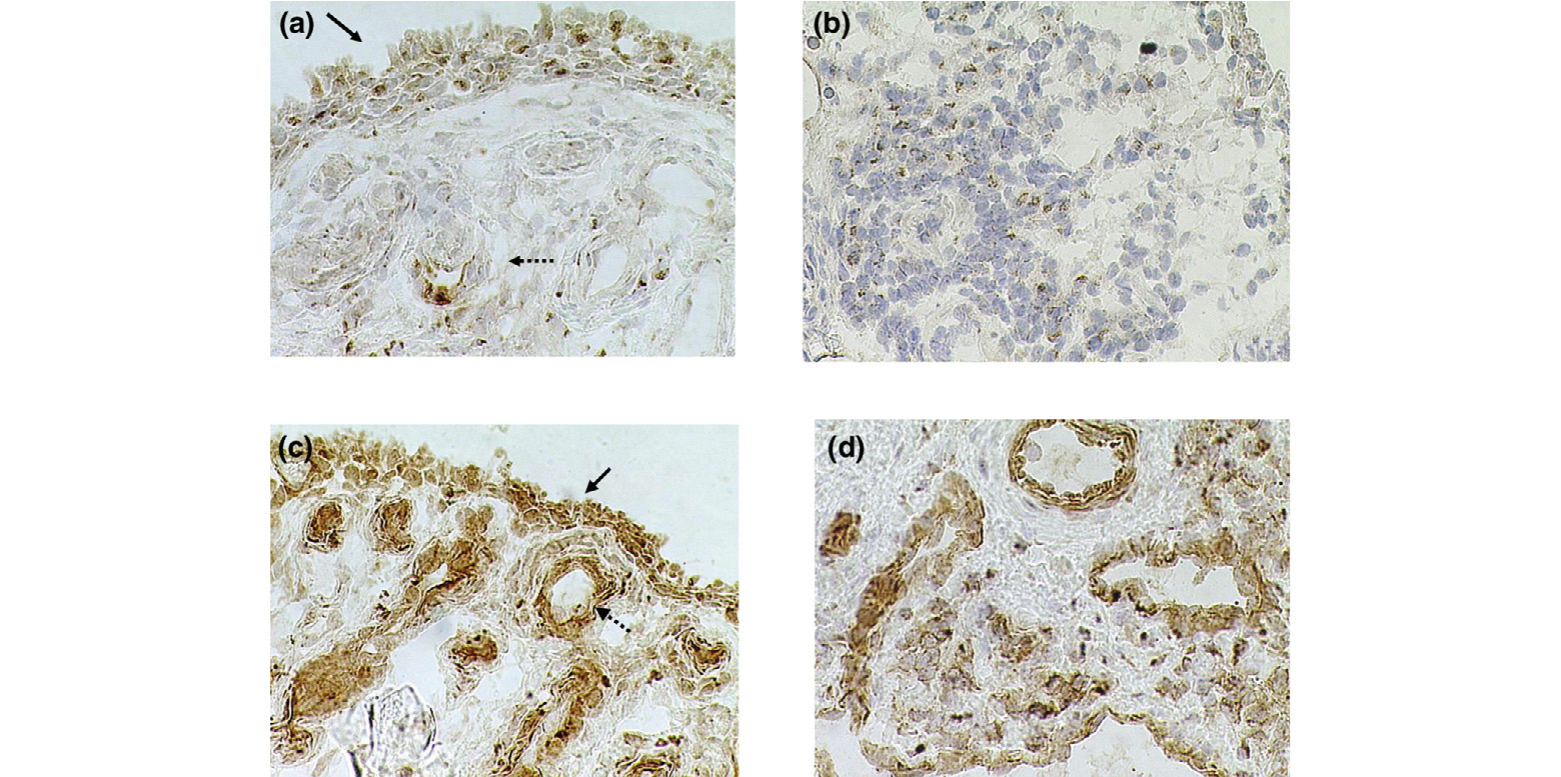
IL-15 is present in synovial tissue in rheumatoid arthritis before and after treatment with infliximab. Sections of synovial biopsy tissue from patient no. 5 show diaminobenzidine staining (haematoxylin counterstained) for IL-15 neutralizing antibody before **(a) **and after **(b) **treatment with infliximab, and for IL-15 non-neutralizing antibody before **(c) **and after **(d) **treatment with infliximab. In (a) and (c) solid arrows indicate synovial lining layer and dotted arrows indicate endothelial cells. Original magnification × 250.

**Figure 2:**
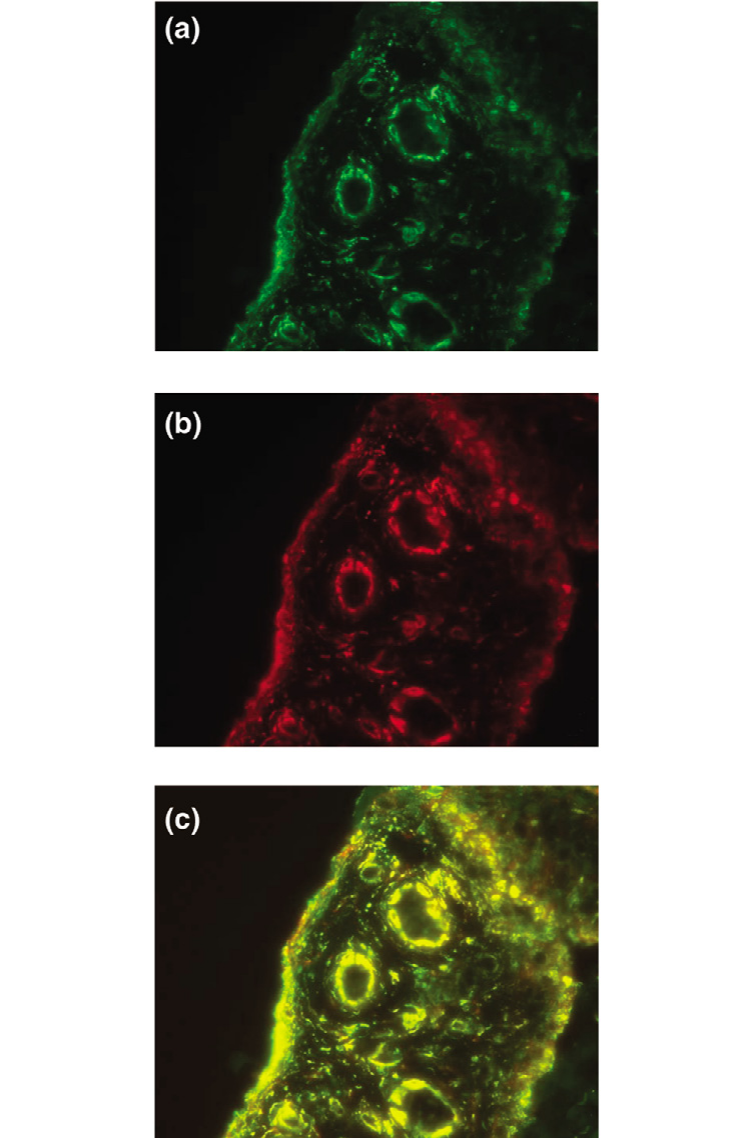
Co-expression of CD163 (macrophages) and IL-15 in the synovial tissue. Immunofluorescence staining of synovial tissue in rheumatoid arthritis of CD163 **(a) **and IL-15 neutralizing antibody **(b) **and double staining with CD163 and IL-15 **(c)**. Original magnification × 250.

IL-15-positive cells were found in the synovial lining and sub-lining layer as well as in endothelial cells (Figure 1) as described previously [3,4]. Positive intracellular staining, indicating IL-15-producing cells, was detected predominantly in the lining layer but also in some endothelial cells (Figure 1a). Double immunofluorescence staining, performed for two of the patients, revealed co-expression between IL-15, analysed with the neutralizing antibody, and CD163 (macrophages) (Figure 2). There was no correlation between the expression of the cytokines investigated and the presence of cells expressing CD3 (T cells) and CD68 (macrophages).

#### Staining for cytokines before and after treatment with infliximab

**Figure 3:**
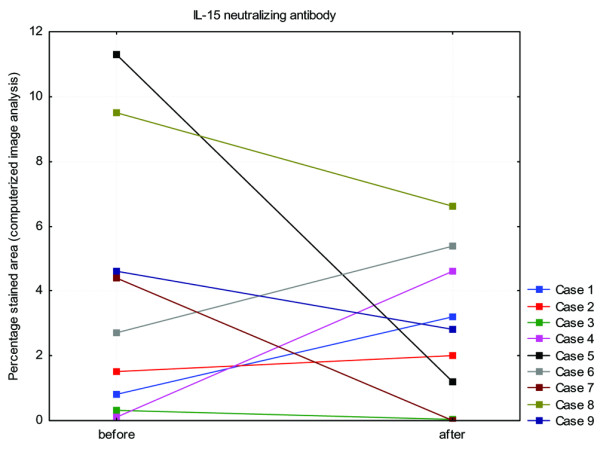
Expression of IL-15 before and after treatment with infliximab. Shown is the expression of IL-15, analysed with the IL-15 neutralizing antibody, in the synovial tissue of nine patients with rheumatoid arthritis before and after treatment with infliximab. The percentage of positively stained tissue area, analysed by computerized image analysis, is presented. No significant difference was observed.

The inter-individual variability in synovial staining was high for the cytokines IL-15, IL-1α, IL-1ß, IFN-γ and TNF, shown in Table 1. The results of both the semi-quantitative analysis and the computerized image analysis of the immunohistochemical staining for different cytokines are shown in Table 2. Positive staining for IL-15 was seen in all synovial biopsies at baseline. After treatment with infliximab, the area that stained positive for the neutralizing anti-IL-15 antibody was increased in four patients and decreased in five (Figure 3). In five patients the area stained with the non-neutralizing anti-IL-15 antibody increased and in three patients the area decreased (data not shown). The number of cells that stained positively with the anti-TNF antibody 2C8 was considerably larger than the number of cells that stained positively with the anti-TNF antibodies mAb1 and mAb11.

### Correlations between clinical parameters and synovial stainings for cytokines

**Figure 4:**
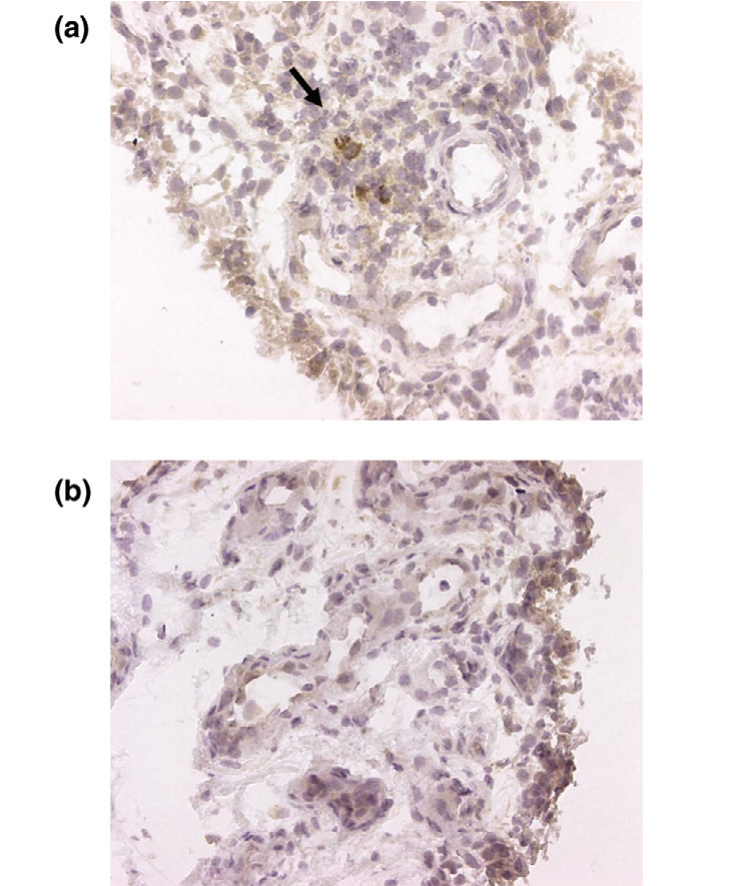
TNF-producing cells in a patient with RA with a subsequent good response to infliximab. Sections of synovial biopsy tissue from patient no. 6 before **(a) **and after **(b) **treatment with infliximab show diaminobenzidine staining (haematoxylin counterstained) for TNF neutralizing antibodies mAb1 and mAb11. Original magnification × 250.

There was no correlation between age, disease duration, health assessment questionnaire and the area or number of cells that stained positively for any of the different cytokine antibodies before and after treatment with infliximab. At baseline, the area that stained positively with the anti-TNF antibody 2C8, analysed by computer image analysis, was significantly correlated with short disease duration (*p *< 0.05). At baseline, the number of cells that stained positively with the anti-TNF antibodies mAb1 and mAb11, analysed by semi-quantitative analysis, was exclusively seen in the patients who fulfilled at least ACR 50% at 3 months (*p *< 0.05) (Fig. 4), whereas all sections from the other patients were negative for these two antibodies.

## Discussion

A major finding in the present study was that the expression of IL-15 in the synovial tissue of patients with RA was not affected by treatment with three infusions of infliximab. IL-15 was detected at baseline in all synovial biopsies; after treatment with infliximab the change in expression diverged, without any correlation with clinical parameters or the expression of other cytokines. Further, we found a co-expression of IL-15 and CD163 when analysed with double staining, indicating that IL-15 in the RA synovial membrane is predominantly produced by macrophages.

The sampling of biopsies was done in a clinical setting, introducing potential bias in the timing of the arthroscopy in relation to the infusion of infliximab. However, previous studies [17,18] indicate that the minor time variations between samplings and infusions in our study did not introduce any bias. Immunohistochemistry with the saponin method is a well established method for detecting the intracellular presence of cytokines by neutralizing antibodies and for further quantification by computerized image analysis. To confirm the staining, a negative control was used for all antibodies. IL-15 production by mononuclear cells, which had been primed to produce IL-15, was entirely blocked by both IL-15 neutralizing and IL-15 non-neutralizing antibodies detecting IL-15, thereby demonstrating the specificity of the staining.

The observation of an overall decrease in synovial cellularity after TNF blockade is consistent with previous studies [14,19-21]. Our finding that the synovial infiltration of mononuclear cells decrease significantly after treatment with infliximab (3 mg/kg), whereas T cells show only a decreasing trend, has also been reported previously [20]. Interestingly, TNF-producing cells, detected by TNF neutralizing antibodies mAb1 and mAb11, were exclusively seen in patients who subsequently responded well to infliximab with at least an ACR 50% response. Although the number of patients studied in this respect was small, the results are in concordance with those of a previous study of shorter duration [14]. In addition, our results on the effects of infliximab on synovial cellularity and the expression of TNF mAb1 and mAb11, IL-1α and IL-1ß are in good agreement with previous published studies [14,20].

It is noteworthy that the results of staining with the TNF-binding antibody 2C8 differed from those obtained with mAb1 and mAb11 in the sense that 2C8 shows rather abundant binding to extracellular material adjacent to cells that are intracellularly stained with this antibody. In addition, more cells are stained with 2C8 than with mAb1 and mAb11, indicating that staining with 2C8 is more sensitive than staining with mAb1 and mAb11, used previously by our group [13-15]. The specificity of the 2C8 antibody for TNF nevertheless seems to be high, because the positive staining was blocked totally by a recombinant TNF. We therefore assume that both sets of anti-TNF antibodies are specific but that they display different sensitivity and possibly also differences in binding to intracellular TNF only (mAb1 and mAb11) or to both intracellular and extracellular TNF (2C8). It is thus of interest in a clinical setting, such as that in the present paper, to describe staining patterns and results for both sets of anti-TNF antibodies. The fact that the mAb1 and mAb11 antibody-derived TNF stainings were able to predict a good clinical response, whereas the 2C8 antibody staining was not, calls for further studies with different methods to quantify TNF and other cytokines in synovial tissues.

The most important finding in the present study is the observation of the presence of IL-15 in synovial tissues both before and after TNF blockade. IL-15 is a pro-inflammatory cytokine with the potential to both induce and maintain inflammation [3,6,7]. The fact that the overall staining for IL-15 did not change during TNF blockade and that IL-15 was present in the inflamed joints of all patients suggests that targeting of IL-15 is an interesting potential therapy in RA irrespective of previous or concomitant therapy with TNF blockade. In a more biological context, the present result also suggests that the IL-15-dependent expansion and maintenance of memory T cells are independent of TNF blockade. This notion is supported by a report in which either TNF or IL-15 could induce the expression of natural killer cell receptor NKG2D on auto-reactive CD4^+^CD28^- ^T cells [22], and that these cells may contribute to the self-perpetuating inflammation in RA. Thus, TNF and IL-15 may, at least in this context, act in parallel and therefore a blockade of TNF can be insufficient as long as IL-15 is present.

## Conclusion

Our study suggests that blockade of IL-15 might provide an attractive treatment as an alternative to TNF blockade in RA, able to function both in patients with low baseline synovial expression of TNF and in patients with an unsatisfactory response to TNF blockade. We also provide additional tentative evidence that the presence and production of TNF in RA joints may be a predictive feature as to whether the patient will respond favourably to TNF blockade.

## Abbreviations

ACR = American College of Rheumatology; DAS28 = Disease Activity Score counted on 28 joints; IFN = interferon; IL = interleukin; mAb = monoclonal antibody; RA = rheumatoid arthritis; TNF = tumour necrosis factor.

## Competing interests

LK has been a clinical investigator in studies of IL-15 blockade (Genmab) as well as in studies of other biologics, including infliximab, etanercept, adalimumab, abatacept and rituximab. He has also served as a consultant/advisor for the companies involved in producing these drugs, including IL-15.AIC has been a consultant to Centocor. The other authors declare that they have no competing interests.

## Authors' contributions

AU, LK and SE designed the study. EaK performed the arthroscopies and synovial sampling. ME sectioned the biopsies and performed the double-stainings. SE, AU and ME developed the immunohistochemical stainings for the expression of IL-15, performed the stainings for cytokines and the semi-quantitative analysis and computerized image analysis of IL-15, TNF and IFN-γ. AIC performed the immunohistochemical stainings, the semi-quantitative analysis and the computerized image analysis of CD markers. ES performed the computerized image analysis of IL-1α and IL-1ß. SE, AU, AIC, EaK and LK prepared the manuscript. All authors read and approved the final manuscript.

## Figures and Tables

**Table 1:** Individual quantification of synovial stainings of cytokines, individual DAS28 values and ACR response rates

Patient	Week	TNF^a^	IFN-γ^a^	IL-1α^b^	IL-1ß^b^	IL-15 neutr.^b^	IL-15 non-neutr.^b^	DAS28	ACR response (%)
1	0	0	1	0.9	0.5	0.8	5.1	6.71	
	9	0	1	0.1	0.8	3.2	13.2	6.34	0
2	0	0	2	2.5	0.4	1.5	3.4	6.00	
	9	0	0	1.2	0.3	2	12.6	4.43	20
3	0	1	1	0.9	7.3	0.3	15.1	7.91	
	9	0	0	0.4	0.1	0	2.1	5.41	50
4	0	0	1	1.2	0.1	0.1	0.6	4.94	
	9	0	2	2.1	33.1	4.6	7.1	4.41	20
5	0	1	1	0.1	0.6	11.3	3.6	5.95	
	9	1	1	2.1	0	1.2	13	2.90	70
6	0	1	1	0.2	2.5	2.7	0.4	7.39	
	9	0	1	0.8	4.4	5.4	7.5	1.79	70
7	0	0	0	0.8	0.7	4.4	2.5	4.83	
	9	0	0	0.1	0.1	0	0	4.23	0
8	0	0	0	2.1	0.5	9.5	16.7	5.64	
	9	0	0	3.3	1.1	6.6	15.1	4.73	0
9	0	0	0	4.9	4.3	4.6	11	5.62	
	9	1	0	3.7	4.4	2.8	6	3.91	20

Individual quantification of stainings of synovial samples from nine patients with rheumatoid arthritis, before and after treatment with infliximab. ^a^A semi-quantitative analysis was performed for tumour necrosis factor (TNF; mAb1 and mAb11) and IFN-γ, for which a semi-quantitative four-point scale was used: 0 = no positive cells, 1 = 1 to 10 positive cells, 2 = 11 to 100 positive cells, and 3 = more than 100 positive cells. ^b^A computerized image analysis was performed for IL-1a, IL-1ß IL-15 neutralizing antibody and IL-15 non-neutralizing antibody; values are median percentages of the stained tissue area, with ranges in parenthesis.

Individual DAS28 values and ACR response results are presented. ACR, American College of Rheumatology; DAS28, Disease Activity Score counted on 28 joints.

**Table 2:** Synovial expression of CD markers and cytokines before and after treatment with infliximab

	Week 0	Week 9	*P*
CD3^a^	2 (0–3)	2 (0–2.5)	Ns
CD68^a^	2.5 (1–3)	1 (0–2)	0.02
CD163^a^	1.5 (0–3)	1.5 (0–2)	Ns
TNF mAb1 and mAb11^a^	0 (0–1)	0 (0–1)	Ns
TNF 2C8^b^	0.25 (0–5.5)	0.3 (0–17.4)	Ns
IFN-γ^a^	1 (0–2)	0 (0–2)	Ns
IL-1α^b^	0.9 (0.1–4.9)	1.2 (0.1–3.7)	Ns
IL-1ß^b^	0.6 (0.1–7.3)	0.8 (0–33.1)	Ns
IL-15 neutralizing^b^	2.7 (0.1–11.3)	2.8 (0–6.6)	Ns
IL-15 non-neutralizing^b^	3.6 (0.4–16.7)	7.5 (0–15.1)	Ns

Synovial expression of CD markers (CD3 (T cells), CD68 (macrophages) and CD163 (fibroblasts)) and cytokines (IL-1a, IL-1ß, IL-15 neutralizing antibody, IL-15 non-neutralizing antibody, tumour necrosis factor (TNF; mAb1 and mAb11) and IFN-γ) was measured before treatment and after a median of 9 weeks of treatment with infliximab in patients with rheumatoid arthritis. ^a^A semi-quantitative analysis was performed for the CD markers CD3, CD68 and CD163. Values are medians of the score for numbers of positive stained cells with ranges in parenthesis; a semi-quantitative four-point scale was used: 0 = no infiltration, 1 = minimal infiltration, 2 = moderate infiltration and 3 = marked infiltration. A semi-quantitative analysis of cytokines, TNF (mAb1 and mAb11) and IFN-γ was also performed. Values are medians of the score for numbers of positive stained cells, with ranges in parenthesis; a semi-quantitative four-point scale was used: 0 = no positive cells, 1 = 1 to 10 positive cells, 2 = 11 to 100 positive cells, and 3 = more than 100 positive cells. ^b^A computerized image analysis was performed for IL-1α, IL-1ß, IL-15 neutralizing antibody and IL-15 non-neutralizing antibody; values are median percentages of the stained tissue area, with ranges in parenthesis. ns, not significant.
